# Influence of Overt Diabetes Mellitus on Cyclosporine Pharmacokinetics in a Canine Model

**DOI:** 10.1155/2009/363787

**Published:** 2009-10-20

**Authors:** Khalid M. Alkharfy

**Affiliations:** Department of Clinical Pharmacy, College of Pharmacy, King Saud University, P.O. Box 2457, Riyadh 11451, Saudi Arabia

## Abstract

*Background/Aims*. Diabetic patients usually require more medications than their nondiabetic counterparts. This work examined the effect of hyperglycemia on the pharmacokinetic properties of cyclosporine in a diabetic dog model. *Main Methods*. Diabetes was induced using a streptozotocin/alloxan combination and verified by measuring the serum glucose level. Cyclosporine was administered as a bolus intravenous dose of 5 mg/kg, and blood samples were collected at different time points for determining drug concentrations and biochemical analyses. *Results*. Diabetic dogs showed a significant increase in total body clearance of cyclosporine compared to healthy controls (0.457 L hr^−1^Kg^−1^ versus 0.201 L hr^−1^Kg^−1^, *P* = .0019) and a decrease in its biological half-life (9.32 hours versus 22.56 hours, *P* = .0125). In addition, diabetic animals exhibited a higher total cholesterol (7.20 ± 0.62 mmol/L and 5.28 ± 0.36 mmol/L; *P* < .05) as well as more serum low density lipoproteins (4.45 ± 0.72 mmol/L versus 1.06 ± 0.10 mmol/L; *P* < .05). *Conclusion*. Overt diabetes alters cyclosporine disposition by modulating its clearance. Abnormalities in the lipid profile, among other factors, may contribute to the accelerated metabolic degradation of cyclosporine under hyperglycemic conditions.

## 1. Introduction

Diabetes mellitus is a complex metabolic disorder that affects a significant fraction of the global population and its management without any side effects is still a major health problem [1–3]. Because of the increased morbidity and mortality of diabetes, more drugs are prescribed to diabetic patients than age-matched nondiabetic people [4, 5]. In many instances, drug therapy in diabetes may involve highly potent drugs or agents with narrow therapeutic ranges. Furthermore, a significant proportion of diabetic patients develop end stage renal failure, and thus, kidney transplantation is not uncommon among them [6, 7]. Diabetes may also ensue following organ transplantation in about 20% of organ recipients because of the immunosuppressive therapy, a condition that is commonly known as post-transplant diabetes [8, 9]. 

It has been proposed that diabetes mellitus may alter the pharmacokinetics of some drugs [10–12]. In essence, diabetes may influence the pharmacokinetic characteristics of drugs by affecting drug absorption–due to gastroparesis or delayed gastric emptying, protein binding–because of glycation of albumin and metabolism owing to differential regulation of drug-metabolizing enzymes [10, 11, 13]. For example, the increased plasma concentrations of triglycerides and free fatty acids due to diabetes can alter the plasma protein binding of drugs [13]. Some investigations have also demonstrated that uncontrolled diabetes in experimental animal models result in enhanced expression of several cytochrome P450 (CYP) isoforms (CYP2E1, CYP2B, CYP3A, and CYP4A) which can be normalized by insulin therapy [13–16]. Fujii et al. reported that chemically induced diabetic mice showed hyposensitivity to pentobarbital manifested by shortening of sleeping time as a result of abnormal hepatic drug metabolism [17]. The metabolism of antipyrine is also increased in patients with type 1 diabetes mellitus, which appears to be due to an increase in CYP1A2 activity [18]. However, changes in drug pharmacokinetics and pharmacodynamics due to diabetes have not yet fully been characterized. Therefore, to improve efficacy and safety of drug therapy in diabetic patients, the effects of hyperglycemia and associated metabolic abnormalities on drug disposition and pharmacological effects require further exploration. 

Cyclosporine A, a lipophilic cyclic polypeptide with immunomodulating properties, has successfully been used for preventing organ transplant rejection [19, 20]. Despite many years of experience, protocols that optimize immunosuppressive drugs efficacy with minimal toxicity remain a subject of debate. Studies of the pharmacokinetic properties of the calcineurin inhibitors, particularly cyclosporine, have led to improved dosing strategies. The therapeutic outcome depends on cyclosporine exposure, which might be characterized by the area under the drug concentration-time curve [21, 22]. Among various factors that influence cyclosporine variability between patients is the concentration of lipoproteins which can modulate cyclosporine binding in plasma (free fraction ~1-2%). Other factors include age, the time posttransplantation, drug interactions, and presystemic elimination by CYP3A or the efflux transporter P-glycoprotein in the gastrointestinal tract [23–25]. 

Due to the adverse effects of cyclosporine on glucose metabolism as well as its potential use in diabetics following kidney/pancreas transplantation, it is of interest to examine the influence of hyperglycemic state on cyclosporine disposition; this is important because little information is available in literature for this effect. Therefore, a series of experiments were conducted to study cyclosporine pharmacokinetics in diabetic dogs and to evaluate diabetes-related factors influencing the variability of cyclosporine blood concentrations.

## 2. Material and Methods

### 2.1. Experimental Animals

Age-matched healthy adult Beagle dogs weighing 8.4–12.8 kg, obtained from the College of Pharmacy Animals Care and Use Facility at king Saud University (Riyadh, Saudi Arabia), were used in the study. Animal were maintained in accordance with the recommendations of the “Guide for the Care and Use of Laboratory Animals” approved by the Facility. They were housed in a temperature-controlled room with a 12-hour light/dark cycle for at least 1 week before the experiments and allowed free water and food *ad libitum* during the study with the exception that the chow was pulled twelve hours prior to animals' dosing. 

Dogs were randomly divided into two groups (*N* = 5 dogs each). Control group was assigned to receive cyclosporine (Novartis, Basel, Switzerland) 5 mg/kg via a slow intravenous (IV) bolus administration. The other group was subjected to diabetes induction using an IV bolus dose of streptozotocin 50 mg/kg and alloxan 50 mg/kg (both obtained from Sigma-Aldrich, St. Louis, MO, USA). Diabetic status was confirmed by measuring fasting serum sugar (FSG) three and ten days following diabetes induction. Those dogs showing FSG of 10 mmol/L or greater were included in the experiments**.**

Treatment with cyclosporine was started ten days after diabetic induction in the beagle dog model with a slow intravenous (IV) bolus of 5 mg/kg through a major leg vein. Blood samples of 1 mL were collected from counterpart leg veins through an indwelling vein catheter into EDTA vacutainers. Blood samples were collected at different time points: 0, 0.083, 0.25, 0.5, 1.25, 1.5, 3, 6, 8, 10, 12, and 24 hours following cyclosporine administration. In all experiments, equal volumes of normal saline were injected through the cannula to replace the fluid loss. Pharmacokinetic and biochemical analyses were carried out on collected samples from diabetic and control dogs.

### 2.2. Cyclosporine and Biochemical Analysis

Measurements of cyclosporine blood levels were carried out using monoclonal whole blood fluorescence polarization immunoassay (TDx system, Abbott Technologies, Abbott Park, IL, USA). Biochemical analysis of some relevant parameters such as kidney function tests (i.e., serum creatinine and blood urea nitrogen) and lipid profile including total cholesterol, high-density lipoproteins (HDL), low-density lipoproteins (LDL), and triglyceride were measured in the plasma of each dog by drawing blood sample just before commencing cyclosporine administration. All biochemical parameters were analyzed by BioSystems kits (BioSystems SA, Barcelona, Spain). The intra- and interday variability of the cyclosporine assay and biochemical analysis were <15%.

### 2.3. Pharmacokinetic Analysis

Pharmacokinetic parameters of cyclosporine were determined by using two-compartmental pharmacokinetic model performed with WinNonlin software (Version 4.1, Pharsight Corporation, Palo Alto, CA, USA). Inspection of semilogarithmic plots of cyclosporine blood concentration-time curves indicated that they could be described by a biexponential decay process. Therefore, the data were initially fitted to one, two, and three compartment models for the best fit. The best fit was based on Akaike criterion and Schwarz's criterion, analysis of residual plots, and correlation matrixes. All blood concentration data were weighted according to 1/*y* ^2^, where *y* is the plasma concentration of cyclosporine. The estimated pharmacokinetic parameters included the intercepts A and B and the macrorate constants *α* and *β*. Additional computed parameters are volume of distribution of the central compartment (V_1_), volume of distribution of the peripheral compartment (V_2_), and the microrate constants (K_12_, K_21_ and K_10_). The noncompartmental analysis was used to estimate the volume of distribution at steady state as (AUMC_0−∞_/AUC_0−∞_)*CL and total body clearance (CL) as dose/AUC_0−∞_, where AUMC_0−∞_ is the area under moment curve and AUC_0−∞_ is the area under the concentration versus time curve from time zero to infinity. AUC_0−∞_ was calculated using the trapezoidal rule as the sum of AUC_0−t_ and any residual area (i.e., extrapolation to infinity) which was computed as the concentration at the last time point by the terminal rate constant. The cyclosporine apparent elimination rate constant (*λ*_z_) was estimated by linear regression analysis of the terminal portion of the log concentration-time data. Cyclosporine apparent elimination half-life (T_1/2*λ*_) was computed as ln 2/*λ*.

### 2.4. Statistical Analysis

Data are presented as the mean ± SEM. Differences in pharmacokinetic parameters of cyclosporine as well as in the biochemical measurements between healthy control and diabetic dogs were assessed by an unpaired *t*-test on log-transformed data. Statistical significance will be assumed when *P* ≤ .05. All calculations were performed using GraphPad Prism version 3.00 for Windows (San Diego, CA, USA).

## 3. Results

Treatment with streptozotocin/alloxan combination induced diabetes in treated animals within 10 days of administration as verified by measuring serum glucose concentration which demonstrated a mean of 24.70 ± 4.63 mmol/L in diabetic dogs versus 6.88 ± 0.69 mmol/L in healthy dogs (*P* = .0003). This hyperglycemic state was also associated with a disturbed serum lipid profile (i.e., dyslipidemia), which manifested as an increase in total cholesterol and LDL levels of 7.20 ± 0.65 mmol/L and 4.45 ± 0.72 mmol/L in diabetic dogs as compared to 5.28 ± 0.36 mmol/L and 1.06 ± 0.10 mmol/L in healthy dogs, respectively (*P* < .05). Other biochemical measures including serum creatinine, blood urea nitrogen, triglyceride, and HDL did not differ significantly between the two groups (Table 1). 

**Figure 1:**
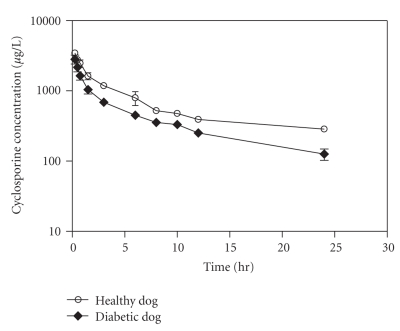
Blood concentration versus time curve of cyclosporine following an intravenous administration of 5 mg/kg dose to healthy and diabetic beagle dogs (*N* = 5 each).

Two-compartment pharmacokinetic analysis of whole blood cyclosporine concentration-time profile of diabetic dogs revealed a significant increase in *α* rate constant of 120% and *β* rate constants of 262% when compared with normoglycemic dogs (Table 2). Also, the rate constant of drug transfer from the peripheral compartment to the central compartment (K_21_) was 336% higher in diabetic dogs than healthy dogs value (*P* = .0186). Noncompartmental pharmacokinetic analysis of cyclosporine concentrations during hyperglycemia also revealed a significant reduction in mean resident time (MRT) and area under concentration-time curve (AUC_0−∞_) amounting to 59% and 52%, respectively (Figure 1, Table 3). Moreover, diabetic dogs demonstrated a significant increase in cyclosporine clearance with a decrease in its biological half-life (0.457 L hours^−1^ kg^−1^ and 9.32 hours) compared to healthy dogs (0.201 L hours^−1^ kg^−1^ and 22.56 hours) (*P* < .05). Cyclosporine's steady-state volume of distribution (V_ss_) was not modified by diabetes in the treated dogs (*P* = .2468).

## 4. Discussion

The present work has demonstrated that diabetic dogs exhibited an altered pharmacokinetic profile of an IV administered cyclosporine as compared to normoglycemic animals. Diabetic dogs showed significant increase in the distribution and elimination of cyclosporine as measured by its disposition half-lives (T1/2*α* and T1/2*β*). In addition, an increase in the rate constant of cyclosporine transfer from the peripheral compartment to the central compartment (i.e., K_21_) has also been observed indicating a significant redistribution of cyclosporine out of tissues. This can increase the availability of cyclosporine for eliminating organs (e.g., liver and kidney) to remove drug faster and therefore shorten its elimination half-life as seen in this study. 

The mechanism of the phenomenon is not entirely understood; however, it could be related to the state of hyperlipoproteinemia that is associated with glucose intolerance. Saad and Najjar reported that streptozotocin-induced diabetes contributes to a disturbed lipid profile [26]. Diabetic dogs in the current study developed a statistically significant higher level of total cholesterol and LDL. In plasma, cyclosporine is predominantly bound to very low-density lipoprotein (VLDL) (10%), LDL (35%) and HDL (33%), and a small remaining portion (10–15%) binds to albumin and globulin [27, 28]. Therefore, hyperglycemia and associated dyslipidemia may play a crucial role in the pharmacokinetic behavior of cyclosporine during diabetes. Interestingly, Aliabadi and colleagues have also demonstrated that hyperlipoproteinemia affects biodistribution of cyclosporine in a rat model. Compared with normolipidemic animals, hyperlipoproteinemic rats had higher plasma, blood, kidney, and liver cyclosporine concentrations [29]. Furthermore, hypercholesterolemia may also attenuate cellular and clinical cyclosporine pharmacodynamics and modulate its uptake by hepatocytes [30, 31]. 

Cyclosporine is a highly protein-bound drug with a low to intermediate hepatic extraction ratio, and thus, its clearance is dependent on the free fraction and intrinsic clearance and possibly blood flow to eliminating organs. Integrity of hepatic metabolic function significantly influences the cyclosporine pharmacokinetics and diabetogenic agents modify differentially and selectively CYP isoenzymes [32]. In fact, diabetes induction by streptozotocin does not change the amount of microsomal protein within the liver, but the hepatic CYP content and enzyme activity are significantly increased [33]. Furthermore, due to the reduced binding of drugs in diabetes, the overall hepatic clearance may also increase leading to enhanced total drug clearance. Diabetic dogs in the current study also exhibited lower serum creatinine values when compared with healthy dogs (54.8 ± 5.92 *μ*mol/L versus 65.6 ± 1.69 *μ*mol/L), which narrowly missed significance (*P* = .0885); this can be attributed, at least partially, to hyperglycemia-induced glomerular hyperperfusion. Although that cyclosporine renal clearance is negligible, increased glomerular filtration rate under such conditions is anticipated to enhance elimination of high renally excreted drugs. 

Therapeutic outcome and severity of expected toxic side effects of cyclosporine are greatly affected by the area under the plasma cyclosporine concentration-time curve [21, 22]. Diabetic beagle dogs treated with cyclosporine showed significant decrease in the MRT and AUC_0−∞_ as well as a great increase in the total body clearance, in addition to dramatic reduction in the elimination half-life of cyclosporine. Given that the volume of distribution of cyclosporine did not change in both treated groups regardless of the pharmacokinetic method of analysis, these observations can be attributed to an increase in the metabolic degradation of cyclosporine in the liver [34]. 

Very little work has been carried out to assess the effect of diabetes on cyclosporine pharmacokinetics, although insulin and glucagon may regulate the metabolizing enzymes and/or transporters involved in cyclosporine disposition [14]. In streptozotocin-induced diabetic rats, D'souza and colleagues showed that cyclosporine clearance was reduced by 127%, indicating that diabetes may profoundly reduce cyclosporine metabolism [35]. Furthermore, insulin administration greatly restored cyclosporine clearance. The findings of the current work, however, demonstrated that overt diabetes significantly increases cyclosporine elimination in beagle dogs. This result is consistent with the observation of lower steady-state average serum concentrations of cyclosporine in pancreatectomized dogs relative to the normals [36]. In addition, diabetic kidney transplant recipients exhibit a higher unbound fraction (fu), higher CL/Fss, and lower AUC values in blood and plasma as compared to nondiabetic patients [37]. Therefore, interspecies differences may exist for the influence of diabetes on cyclosporine pharmacokinetics, and further work is warranted to evaluate the mechanism(s) of these observations. Interestingly, readjustment of the insulin administration normalized the drug metabolism as has been seen in experimental studies [38, 39] which highlights the importance of proper glycemic control to minimize the influence of diabetes on drug disposition.

## 5. Conclusion

The current study has demonstrated that diabetes modifies the pharmacokinetic profile of cyclosporine in beagle dogs. Increased total body clearance due to hyperglycemia and/or associated disturbed lipid profile could be the basis of this modulation. More studies are needed to further characterize the effects and mechanisms of diabetes on the pharmacokinetics and pharmacodynamics of other clinically used drugs.

## Figures and Tables

**Table 1:** Biochemical parameters in healthy and diabetic beagle dogs.

Parameter	Group	Mean ± SEM	*P* value
Fasting serum glucose (mmo/L)	Healthy	6.88 ± 0.69	.0003
	Diabetic	24.7 ± 4.63	
Total cholesterol (mmo/L)	Healthy	5.28 ± 0.36	.0279
	Diabetic	7.2 ± 0.65	
High-density lipoprotein (mmo/L)	Healthy	3.38 ± 0.25	.8331
	Diabetic	3.31 ± 0.28	
Low-density lipoprotein (mmo/L)	Healthy	1.06 ± 0.1	.0001
	Diabetic	4.45 ± 0.72	
Triglyceride (mmo/L)	Healthy	0.73 ± 0.14	.5042
	Diabetic	0.96 ± 0.23	
Serum creatinine (*μ*mo/L)	Healthy	65.6 ± 1.69	.0885
	Diabetic	54.8 ± 5.92	
Blood urea nitrogen (mmo/L)	Healthy	4.0 ± 0.85	.6719
	Diabetic	4.3 ± 0.66	

**Table 2:** Compartmental pharmacokinetic parameters of cyclosporine following an intravenous administration of 5 mg/kg in healthy and diabetic beagle dogs.

Parameter	Estimate (Mean ± SEM)		
	Healthy dog	Diabetic dog	*P* value
A (*μ*g L^−1^)	3676.57 ± 857.15	2866.58 ± 419.88	.4237
B (*μ*g L^−1^)	553.77 ± 92.76	738.09 ± 79.93	.1706
*α* (hr^−1^)	0.74 ± 0.28	1.63 ± 0.36	.0477
*β* (hr^−1^)	0.026 ± 0.008	0.094 ± 0.026	.0207
K_12 _(hr^−1^)	0.53 ± 0.25	0.89 ± 0.22	.1404
K_21 _(hr^−1^)	0.11 ± 0.022	0.48 ± 0.16	.0186
K_10 _(hr^−1^)	0.13 ± 0.03	0.34 ± 0.07	.1076
V_1_ (L kg^−1^)	1.35 ± 0.16	1.49 ± 0.22	.5669
V_2_ (L kg^−1^)	6.95 ± 2.86	3.04 ± 0.38	.1788

**Table 3:** Noncompartmental pharmacokinetic parameters of cyclosporine following an intravenous administration of 5 mg/kg in healthy and diabetic beagle dogs.

Parameter	Estimate (Mean ± SEM)		
	Healthy dog	Diabetic dog	*P* value
C_o_ (*μ*g L^−1^)	3680.50 ± 661.58	3721.28 ± 638.62	.8801
MRT (hr)	25.73 ± 3.04	10.6313 ± 2.35	.0098
AUC_0−t_ (*μ*g*hr L^−1^)	15892.66 ± 1225.49	10144.39 ± 1113.72	.0119
AUC_0−∞_ (*μ*g*hr L^−1^)	25181.39 ± 1513.63	12110.47 ± 1881.94	.0019
CL (L hr^−1^ kg^−1^)	0.201 ± 0.010	0.457 ± 0.076	.0019
V_ss_ (L Kg^−1^)	5.12 ± 0.57	4.19 ± 0.45	.2468
*λ*z (hr^−1^)	0.032 ± 0.003	0.106 ± 0.037	.0125
T_1/2*λ*_ (hr)	22.56 ± 2.3042	9.3194 ± 2.2896	.0125
